# Studies of Vision in Cricket—A Narrative Review

**DOI:** 10.3390/vision7030057

**Published:** 2023-08-28

**Authors:** Jennifer Swingle Fogt, Nick Fogt

**Affiliations:** College of Optometry, The Ohio State University, Columbus, OH 43210, USA; fogt.4@osu.edu

**Keywords:** vision, cricket, visual training method, optometry, motor skills, sports vision

## Abstract

Vision is thought to play a substantial role in hitting and fielding in cricket. An understanding of which visual skills contribute during cricket play could inform future clinical training trials. This paper reviews what has been reported thus far regarding the relationship of visual skills to cricket performance and reviews the results of clinical trials in which the impact of visual skills training on cricket performance has been addressed. Fundamental or low-level visual skills, with the exception of color vision and perhaps near stereopsis and dynamic visual acuity, are similar between cricket players and the general population. Simple reaction time has been found to be shorter in cricket players in some but not all studies. While there is mixed or no evidence that the aforementioned visual skills are superior in cricket players compared to non-players, comparisons of eye and head movements and gaze tracking have revealed consistent differences between elite cricket batters and sub-elite batters. Future training studies could examine whether teaching sub-elite batters to emulate the gaze tracking patterns of elite batters is beneficial for batting. Lastly, clinical trials in which visual skills of cricket players have been trained have in many cases resulted in positive effects on visual skills, or judgments required in cricket, or cricket play. However, clinical trials with larger and more diverse groups of participants and correlations to on-field metrics and on-field performance (i.e., domain-specific assessments) are necessary before conclusions can be drawn regarding the efficacy of vision training.

## 1. Introduction

A wide array of vision-related attributes are likely to play a role in cricket. A number of investigators have proposed schemes by which to group visual skills that pertain to sports. For example, Ciuffreda and Wang [1] listed five major categories for these visual skills including the following:

1. Resolving detail;
2. Estimating depth;
3. Tracking moving objects;
4. Visuomotor integration;
5. Visual information processing.

Other investigators have proposed similar schemes, including Kirschen and Laby [2] and most recently Hodges and colleagues [3]. Applying the categories of Ciuffreda and Wang to cricket, for example, one could categorize a cricket batter‘s ability to resolve details such as kinematic cues from the bowler’s motion and the rate of increase in the retinal image size of the approaching ball as skills related to “resolving detail”. Similarly, estimating depth pertains to the rate of change in retinal disparity of the approaching ball when batting. A batter can track the approaching ball by employing eye and head movements, which may help in estimating the ball’s speed and trajectory. In terms of visuomotor integration, in order to strike the approaching ball, a cricket batter must perform a series of coordinate transformations that ends with the bat in the proper location to intercept the ball [4]. Finally, in terms of visual information processing, a cricket batter may anticipate when and where the approaching ball may arrive based on kinematic cues associated with the bowler’s motion, or based on previous experiences, or based on “situational knowledge” such as knowledge of a bowler’s tendencies or the placement of the fielders [5].

The goal of sports vision is to determine which visual skills influence sports performance, and to train those visual skills to maximize performance during competition. From the categorization schemes described above, one could first develop a list of specific visual skills that may impact cricket batting and fielding performance, and then indirectly (ex situ or laboratory assessments) or directly (in situ or on-field assessments) determine whether these visual skills could or do influence in-game performance.

There are multiple methods that have been used to determine which visual skills may be important for a particular sport. One of these methods is to compare a particular visual skill between expert performers and novice or less-expert performers. If a visual skill is shown to be better in experts, this could imply that this skill plays a role in performance, requiring further sport-specific study of the skill. A second method is to compare or correlate the visual skill with performance metrics from competitions. This method only indirectly suggests a relationship between the visual skill of interest and performance in competition. This is because regression analyses correlating visual skills and performance reflect associations between these variables, but they do not necessarily imply that visual skills lead to better performance. Finally, a third method to assess the impact of a visual skill on sport performance is to perform a placebo-controlled clinical trial. In this scenario, a visual skill or group of skills are trained. Pre-training and post-training assessments of either visual skills thought to be related to sports performance or of skills directly related to performance may be compared. Ideally, the outcome of clinical trials would include a comparison of pre-training and post-training on-field performance metrics. Clinical trials involving pre-training and post-training measures of game-related skills (e.g., batting or fielding) are most definitive in establishing the efficacy of the training paradigms.

Hodges and colleagues divided visual skills for interceptive sports into fundamental skills (visual acuity, visual field), low-level visual skills (color vision, contrast sensitivity, stereoacuity, motion perception), high-level visual and attentional skills (visual attention, eye movement control), and cognitive skills (anticipatory decisions, general decision making, memory, situational knowledge, and general executive functions). Under each of these skills, it is possible to draw a distinction between domain-specific and domain-general training methods and assessments [3]. In this paper, domain-specific methods are defined as those that use the stimuli and require responses similar to those in a cricket match (e.g., predicting the trajectory of an approaching ball or playing a shot) [6]. On the other hand, domain-general methods are those that typically use stimuli and require responses that are not specific to cricket (e.g., visual acuity or ocular alignment). While these definitions will be adhered to throughout this paper, it should be noted that another factor that has been used to differentiate between domain-specific and domain-general assessments is the environment in which visual skills are assessed. That is to say, some investigators have described domain-specific methods as including not only the stimuli and responses required in competition, but the “on-field” conditions as well [6]. In this paper, we do not include the environment in differentiating domain-specific and domain-general methods, although it has been argued that those sports vision training methods that are most likely to transfer to improvements in on-field performance are highly domain-specific and include the stimulus, the response, and the environment found in the actual competition [7,8].

For the various categories of skills proposed by Hodges et al., both domain-specific and domain-general methods have been used to compare athletes with different skill levels [3]. While some of these studies reported significant differences in responses, others did not. Currently, the question of whether domain-specific approaches are more likely to reveal differences when expert athletes are compared to less-expert or novice athletes has not been answered.

Because there are published studies on vision in cricket that encompass both low-level and high-level visual skills as well as domain-specific and domain-general visual skill assessments, the sport of cricket provides an interesting opportunity to examine questions such as whether one group of skills or one group of assessments (low-level or high-level) correlate better with the skill level of players in the context of just one sport. The focus on one sport is significant, since it is unclear whether the assessment and training of visual skills in one sport transfers to another sport [7,8].

In this narrative review, we first summarize studies in which the visual skills of cricket players at different levels are compared and studies in which the visual skills of cricket players and the general (non-playing) population are compared. The purpose of this first section of the paper is to determine which visual skills have thus far been shown to be better in cricket players, to describe the method of assessment of these skills (domain-specific or domain-general), and to determine whether particular groups of visual skills (e.g., lower-level skills such as visual acuity or higher-level perceptual-cognitive skills) are more likely to be different for cricket players at different levels or in cricket players versus non-players [9].

In the second section of the paper, we review studies in which the impact of vision training on cricket performance is assessed. This part of the review includes 12 studies, which to our knowledge represents the most comprehensive review on this topic in cricket thus far. The visual skills trained in each of these twelve studies will be compared to the visual skills shown to be important for cricket performance in the first section of the paper to determine whether the training paradigms are in fact targeting skills that are expected to enhance performance. Then, the outcome measures of each training study will be evaluated to determine if they are domain-specific or domain-general measures. The review will inform future studies on vision in cricket.

## 2. Methods

Papers used in the following sections were identified using four sources. The first source was PubMed (National Library of Medicine), the second source was Google Scholar, and a third source was SPORTDiscus. The keywords “Cricket” and “Vision” were used in all of these searches. Finally, a general search was performed using the “Google” search engine. This latter search was primarily executed using the search term “vision in cricket”. Reference lists in the publications yielded through these searches were also examined for relevant papers and book chapters. The majority of these resources were research articles.

## 3. Comparisons between Players and Non-Players and between Players at Different Levels

### 3.1. Cues to Trajectory

To begin the discussion of those visual skills that have been compared between cricket players and non-cricket players or between cricket players at different levels, in this section, we discuss those non-visual and visual cues that cricket players may use to intercept projected balls. Much of our understanding of these cues is derived from temporal occlusion studies, in which the vision of the observer is occluded at different times prior to and after pitch release [10]. Spatial occlusion studies in which a portion of the visual scene is obscured from the observer have also been performed [11]. These occlusion studies all involve domain-specific assessments.

#### 3.1.1. Advance Cues

Advance cues, which include both contextual and kinematic cues, are those cues available prior to release of an approaching ball. Contextual cues are sources of information about a projected ball’s speed or trajectory that are known to the batter or fielder prior to any action on the part of a bowler or batter. These cues can help in predicting the trajectory of the ball. In the case of cricket batters, examples of contextual cues could be the batter’s knowledge of a bowler’s tendencies, or the batter’s knowledge regarding the most likely ball types associated with the current placement of the fielders [7]. Kinematic cues are associated with a bowler or thrower’s pattern of motion. Collectively, studies using occlusion have led to a two-stage model of interceptive behavior formulated by Müller and Abernethy [12] and elaborated on by Morris-Binelli and Müller [7] in which contextual and kinematic information is used to “position” the lower body (foot movements and weight transfer), and ball flight information “fine-tunes” the interceptive action (bat–ball contact or a check-swing). In cricket batting, for example, Müller and Abernethy used the occlusion technique to compare the use of kinematic and ball flight cues between six “highly skilled” cricket batsmen from the Australian Cricket Academy and six “low-skilled” players who had no cricket experience above the junior (under-16) level [5]. The investigators concluded that neither group made effective use of advance cues in isolation to guide foot placement or to produce efficient bat–ball contact. On the other hand, the highly skilled players were able to more efficiently use ball flight information after the ball was released and prior to ball bounce to achieve bat–ball contact. In a follow-up study, Müller and colleagues again used the occlusion technique to assess those cues used by cricket batters [13]. Subjects included six (“highly skilled”) batsmen from the Victorian State Cricket (First-Class) Squad and six (“low skilled”) university students who had not played above the junior (under-16) level. For full-length deliveries, foot placement was improved for highly skilled players compared to low-skilled players when (early) trajectory information from ball flight was available. For short-length deliveries, highly skilled players used advance cues more effectively than low-skilled players for foot placement. In the same study, for full ball lengths, Müller and colleagues concluded that ball flight information was the primary determinant of bat–ball contact for both highly skilled and low-skilled players [13]. Highly skilled players were also more effective in using ball flight information after ball bounce to increase the number of “good” bat–ball contacts. Müller et al. pointed out that this suggests that early ball flight informs “initial” bat positioning, while post-bounce information is used to “fine tune” the swing. For short-length balls, it was similarly concluded that highly skilled players made better use of early flight information to contact the ball, and highly skilled players made use of ball bounce and post-bounce information to increase the number of good bat–ball contacts.

#### 3.1.2. Integration of Contextual and Kinematic Cues

Müller and colleagues have also examined the integration of contextual and kinematic cues [14]. In one study, skilled cricket male batters from the Trinidad, Barbados, and Jamaica Cricket Associations were assessed [10]. The investigators concluded that these batsmen did not make use of kinematic information in predicting the type of ball (full outswinger that curved right, full inswinger that curved left, and short ball that bounced higher) thrown by a bowler and instead relied on contextual information. In another study, Müller and colleagues tested 24 male batsmen from Australian State Cricket Association elite squads [14]. In this group were 6 first-class players, 10 individuals who were part of the state under-19 team, and 8 individuals who were part of the state under-17 team. Participants viewed videos of a bowler who bowled three different ball types (full-length outswinger, full-length inswinger, and short-length delivery). At various times, the participants’ vision was occluded. In addition, contextual information (game-specific progressive score and fielding placings) was provided that was either congruent or incongruent with the ball type. The participants’ task was to anticipate the ball type. Some of the more important conclusions were that the first-class batsmen began integrating contextual cues and kinematic cues earlier than the other participants. However, anticipation in all three groups of participants was negatively influenced by late kinematic information when that information was incongruent with contextual information. These results again suggest that contextual information is prioritized over kinematic information. The investigators concluded that occlusion training should be instituted with cricket batters in order to improve batter’s attention to and integration of contextual and kinematic cues. This latter conclusion follows from Morris-Binelli and Müller’s two stage-model for interception in sports [7]. In the first stage of this model, expert anticipation depends on the player integrating earlier contextual cues with later kinematic cues [14]. In a different study relating contextual cues to kinematic cues, Sarpheshkar and colleagues examined the behavior of cricket batters when swinging at only straight (linear) bowl trajectories and when swinging at randomly presented straight and curved (curvilinear) bowl trajectories [15]. These investigators demonstrated that the possibility of the ball curving, which represents a contextual cue, was enough to change the batters’ behaviors for the straight trajectories. Specifically, in batting the straight trajectories when these trajectories were randomly mixed with curved trajectories, batsmen played more front-foot defensive shots (i.e., the batter moves forward and minimizes the swing follow-through) and batters hit the ball further forward compared to when there was no possibility of a curved trajectory. This suggests that, at least to an extent, kinematic and ball flight cues were superseded by contextual cues. Changes in the batters’ kinematics, however, did not significantly affect the percentage of good bat–ball contacts.

#### 3.1.3. Ball-Flight Cues

Müller and Abernethy suggested that the trajectory information available from a ball’s flight included the rotation, “apex”, and “drift and dip” of the approaching ball [5]. Regan has described the optical cues associated with an approaching ball that are available in the retinal array that may be used to judge when and where a cricket ball will arrive [16]. These cues presumably contribute to performance in interceptive sports [17]. In terms of binocular cues, the passing distance of an approaching object can be determined from the velocity of the ball’s binocular image and the rate of change in the horizontal disparity of the ball. Interestingly, Regan mentions studies in which estimates of passing distance were found to be best when the direction of motion in depth was “midway” between the eyes, and the author suggests that maintaining one’s head over the ball could be beneficial in cricket batting. Regan also proposed an equation for monocular estimates of passing distance.

To estimate the time at which an approaching object will reach an observer, the ratio of the object’s instantaneous angular subtense to the rate of change in the ball’s angular subtense (termed tau) might be used. A binocular ratio to estimate the time of arrival of an approaching object has also been derived, and it involves the rate of change in the object’s horizontal disparity.

Given that there is detailed visual information associated with a cricket ball’s flight that can be used in estimating the ball’s trajectory, a reasonable suggestion would be that low-level visual functions such as visual acuity could impact on cricket performance. However, if characteristics such as making better use of contextual cues or early kinematic cues or more accurately directing attention to locations where the most specific information related to ball trajectory are found have a greater influence on cricket performance compared to low-level visual skills, then low-level skills may not need to be any better in cricket players than in the general population. The following section looks at what is known thus far about the impact of low-level visual functions on cricket performance.

### 3.2. Fundamental and Low-Level Visual Functions

#### 3.2.1. Eye Examination Findings

As mentioned above, Hodges and colleagues recently published an extensive scheme to classify perceptual-cognitive skills involved in interceptive sports [3]. Fundamental and low-level visual skills in this scheme include visual acuity, visual field, color vision, contrast sensitivity, stereoacuity/depth perception, and motion perception/sensitivity. One might expect that better low-level visual skills would result in better on-field performance in cricket. For example, clear vision and dynamic stereopsis would help in assessing kinematic cues and optical cues for hitting and fielding. In studies on cricket, these low-level skills have generally been assessed in a domain-general manner by comparing the visual skills of athletes to the wider population [18,19]. A variant of this approach has also been employed in which visual skills of players at higher and lower levels are compared.

The literature search identified four papers in which fundamental and lower-level skills in cricketers were studied using domain-general assessments. Barrett and colleagues examined two groups of cricketers and a group of rugby players [9]. The focus here will be on the cricket players. For the cricketers, one group consisted of 23 male “near-elite” cricket players from universities in England. Some of these players had competed with English county teams. The second group consisted of 16 elite female players from England’s international women’s cricket team. Static visual acuities (logMAR) at distance and near were assessed, as was the percentage of individuals in each group that showed improvement in the distance visual acuity in either eye when viewing through a pinhole. Stereoacuity (TNO stereotest) and color vision (Ishihara 24-plate edition) were also assessed. Finally, participants were asked questions including but not limited to the regularity of their eye examinations and their use of spectacles and contact lenses.

The results for tests of visual function were compared to previously published values on young adults. Overall, distance visual acuity in the cricket players was no better than that published for young adults, and for the elite cricketers, visual acuity was statistically worse than that expected from published values. One of the 16 elite players and 2 of 23 near-elite players showed improvement in distance acuity greater than or equal to one line with the pinhole, suggesting that there was uncorrected refractive error for these players. Near visual acuity was also similar to that expected from the published literature. For the near-elite cricket players, stereoacuity was similar to the expected value while stereoacuity was better than expected in the elite cricket players. Color vision was normal except for two near-elite cricketers. Other visual problems, referred to by the authors as “residual visual issues” were found in three (18.8%) of the elite cricketers and eight (35%) of the near-elite cricketers. A number of these residual issues were due to uncorrected refractive error, or because players did not wear their refractive correction during games. Of the 16 elite cricketers, 69% (11) had received an eye examination within the last two years, 25% (4) had received an examination within 2–5 years, and 6% (1) had received an eye examination more than 5 years ago or never. Of the 23 near-elite cricketers, 52.2% (12) had received an eye examination within the last two years, 17.4% (4) had received an examination within 2–5 years, and 30.4% (7) had received an eye examination more than 5 years ago or never, suggesting that the rate of eye care utilization was modest.

Overall, Barrett and colleagues concluded that the fundamental and low-level visual skills they assessed were not generally better than those expected in the population except for near stereoacuity in the elite players, and that perfectly clear vision as measured clinically is not required for successful performance in cricket or rugby. Much like other sports vision researchers have concluded [20,21], the results suggest that what may differentiate skilled and less skilled athletes are higher-level perceptual-cognitive processes such as more rapid and more effective anticipation.

In a second study of fundamental and low-level visual skills in cricket players, Brown and Couper examined 100 cricket players at different levels, some who played for district clubs and some who played for sub-district clubs [22]. All of the assessments were domain-general. The participants (presumably all male) ranged in age from eighteen to thirty-four years of age. Static far and near (corrected) visual acuities, oculomotor alignment, smooth pursuit eye movements, saccadic eye movements, convergence eye movement, stereoacuity, and color vision were all assessed. The results for the players were compared to normative values from two previously published studies. Most of the players (95%) had 6/6 or better vision in both eyes. The authors concluded that the percentage of cricket players in their study with strabismus (4%) and heterophoria (40%) was similar to that reported in the literature, and the other oculomotor tests also “showed no significant abnormalities” compared to the previously published studies. Similarly, the percentage of individuals with a stereoacuity threshold of 60 s of arc or better was said to be similar to other studies. Finally, 3% of the study participants were color deficient, less than the 8% population value for males. The authors also compared the test results between players at higher and lower grades, concluding that in general, there was no relationship between a particular vision test and the grade level of the player except that there was a higher rate of orthophoria in players at higher grades. While visual acuity levels in their participants were perhaps, in the authors’ own words, “better than average”, these investigators pointed out that there was no correlation between visual acuity and cricket grade level. Although not directly stated, the authors’ comments suggest that visual acuity was not considered a determinant of performance. On the other hand, the higher rate of orthophoria in the higher grades could suggest that convergence training may be useful for cricket players, although the amount of heterophoria was not specified and could have been clinically insignificant.

In a third study of fundamental and low-level vision in athletes, Sapkota and colleagues examined 95 national football (soccer) and cricket players from Nepal [23]. All of the individuals who were examined had represented Nepal in at least one international tournament. A total of 18 of the 95 players were cricketers. Twenty percent of the study participants were female, and all of these individuals were football players. Sixty nine percent of the players had never had a complete eye examination, demonstrating once again that many athletes do not regularly visit an eyecare professional. Ocular “problems” were diagnosed in 76.8% of the players, including exophoria in 23%, pinguecula or pterygium in 21%, refractive error in 8.5%, and ocular injury in 8.5%. Overall, these data suggest that ocular issues occur with regularity in athletes and that these issues do not necessarily preclude elite athletic performance.

Finally, in a fourth study involving low-level skills in cricket players, Kelly and Roberts compared the dynamic visual acuity of male cricket players to male non-cricket players [24]. Dynamic visual acuity as tested in this study involved detecting the position of the gap in a moving Landolt-C ring. Participants included eight sub-elite varsity-standard cricket players and eight participants who had no competitive experience in cricket. The Landolt-C ring was moved horizontally or vertically on a screen at angular velocities of 15 deg/s, 9.15 deg/s, or 3.06 deg/s. The size of the ring was increased until the observer could determine the location of the gap in the Landolt-C. For both the players and non-players, dynamic visual acuity declined as the stimulus velocity increased. However, the relative reduction in acuity with increasing velocity was less for the players compared to the non-players.

While such differences in dynamic visual acuity between athletes and nonathletes have also been documented in other studies [25,26], in another study, Edgar and colleagues found no significant differences in the dynamic visual acuity of 13 male cricket players, some of whom were from the Auckland under-19 winter development training squad and some of whom were simply known to play cricket, compared to 18 male “non-cricketers” [27]. While it is difficult to determine whether differences in the skill level of the cricket players and non-players who participated in these studies can account for the discrepant findings of Kelly and Roberts and those of Edgar, it should be noted that the participants in the study of Edgar et al. were given less time to observe the stimulus compared to the study of Kelly and Roberts. Reduced observation time would therefore have limited the time for tracking eye movements in the study of Edgar et al., and this is known to decrease differences in dynamic visual acuity between athletes and non-athletes [25]. The relationship between eye movements and cricket performance will be expanded upon later in this paper.

#### 3.2.2. The Impact of Visual Blur

In agreement with the domain-general assessments in the studies described above [9,22,23], studies in which domain-specific responses were required also suggest that perfect vision is not required in cricket. In a series of papers, Mann and colleagues examined the influence of blurred vision on cricket batting. In the first of these papers, these investigators examined batting for eleven male subjects [28]. The subjects were either players of clubs in the Sydney Grade Cricket Competition or those who had participated in the last five years as “a junior state or first grade country representative”. Subjects batted balls projected by a cricket bowling machine at speeds of 105–113 km per hour (65–70 miles per hour). The trajectory of each ball was varied. Subjects batted while wearing plano, +1.00 D, +2.00 D, and +3.00 D contact lenses. The plus lenses blurred the subjects’ vision. A cricket coach evaluated the quality of the shots on a scale from 1–10 by viewing videos of the batters’ performance. This subjective score was based on the appropriateness of shot selection and shot execution. Batting performance was mostly unaffected (compared to the plano contact lens condition) with the +1.00 D and +2.00 D contact lenses, and performance only significantly declined when the +3.00 D contact lenses were worn.

In a second paper, Mann and his collaborators pointed out and addressed some issues with their original study [29]. First, the original study made use of a bowling machine, which eliminates kinematic information from the bowler’s motion prior to ball release and which has a more consistent release point than real bowlers are likely to have. The influence of blur may therefore be less when batting balls from a bowling machine compared to batting balls from a real bowler. Second, the speed of the projected balls in the original study was relatively low, and the authors proposed that blur may have more impact on batting when balls are projected at higher speeds. Finally, the scoring system for shot quality in the original study may not have been sensitive enough to detect changes induced by blur. Ten male cricket batters who had played in the “local regional first-grade competition” in the last 12 months participated as subjects. A similar experimental paradigm to that of the first study was used, in that viewing conditions of plano, +1.00, +2.00 D, and +3.00 D were employed. However, while a bowling machine was used in one condition, in the other condition, live bowlers were used. Two of these bowlers bowled the ball at speeds between 90 and 110 kph (56–63 mph) (similar to that of the bowling machine and similar to the speed used in the original study, and called “medium pace”), and the other bowled the ball between 120 and 130 kph (75–81 mph) (faster than the speed used in the original study, and referred to as “fast pace”). The trajectory of the projected balls with both the bowling machine and the live bowlers was randomly varied. Shot quality was assessed using three categorical variables: QoC (quality of bat–ball contact), FoBs (forcefulness of bat-swing), and LoD (likelihood of dismissal). For both the bowling machine and the live bowler conditions, overall performance was not affected until +3.00 D of blur was induced. However, the performance for the fast deliveries (120–130 kph) declined significantly at +2.00 D.

Finally, Mann and colleagues published a study in which ten male cricket batters who had played in the “local regional first-grade competition” within the 12 months prior to the study viewed balls bowled by live bowlers [30]. The participants’ vision was either occluded near the time that the bowler released the ball or was not occluded. Only those data from the occlusion condition were analyzed. The participants were required to either make a verbal judgment (uncoupled response) of the ball’s direction (either toward the batter’s legs or away from the batter’s legs) or to hit the ball (coupled response) with a bat. Once again, batters’ responses were made while viewing through contact lenses with powers of plano, +1.00 D, +2.00 D, and +3.00 D. In the plano lens-wearing condition, performance was better when subjects attempted to hit the ball (coupled condition) compared to when subjects made a verbal judgment regarding the ball’s trajectory (uncoupled condition). The performance in the plano viewing condition was above the chance level in the coupled condition, but it was below chance for the uncoupled condition. In the coupled condition, consistent with the two studies by Mann and colleagues described above, performance was unaffected by +1.00 D and +2.00 D of blur but did decline with +3.00 D lenses. Interestingly, uncoupled responses improved with blur, a finding consistent with other studies [21]. The use of blurred stimuli for training in sports related tasks is an active area of research [21].

Taken together, the results of these three studies suggest that blur of as much as +2.00 D has little influence on performance in hitting balls in cricket. These results are somewhat similar to those in baseball, where Reuscher and colleagues showed that the performance of batters hitting balls from a pitching machine was not affected with +1.00 D of blur, but performance was reduced with +2.00 D of blur [31].

The fact that blur less than +3.00 D does not negatively impact on cricket batting at least for slow to medium speed deliveries (less than 110 kph) might suggest that batting is largely regulated by the dorsal visual system (the vision-for-action system) rather than the ventral visual system. The dorsal visual system is said to be relatively insensitive to blur [32].

While the conclusion from Barrett and colleagues’ visual acuity assessments (domain-general) and from Mann and colleagues’ studies of blur and visual performance (domain-specific) is that perfect vision is not necessary to successfully bat a cricket ball, Mann and colleagues make the point that these results do not argue against providing the “full” refractive correction for cricket players. What the results do suggest is that blur resulting from uncorrected refractive error may not negatively impact on performance as much as one might expect.

#### 3.2.3. The Visual Field and Batting Helmets

Another fundamental visual skill related to athletic performance mentioned by Hodges and colleagues is the visual field. A study by Wilkins and colleagues regarding the visual field of cricket batters should be mentioned here [33]. Although this study does not involve a comparison of the visual field of cricket players and the general population or a comparison of the visual field between cricket players at different levels, it does demonstrate how the visual field may impact upon cricket performance. The authors explain that new standards related to batting helmets that are intended to protect the batter from eye and facial injuries have resulted in a reduction in the gap between the peak and grille of the helmet. The gap between the peak and the grille must be smaller than the diameter of the ball to meet these new standards. Wilkins et al. assessed the monocular visual field of 10 amateur male cricket players using a Humphrey Visual Field Analyzer (Zeiss, Oberkochen, Germany) both while the participants wore the old style (prior to the new standards) helmet and the new helmet (intended to conform to the new standards). The authors concluded that the superior visual field was reduced to a greater extent than the other fields with the new helmet compared to the old helmet. While the superior field (the portion of the superior visual field about 25–30 degrees from fixation) was primarily reduced with the new helmet, the inferior field (the portion of the inferior visual field between about 25–30 degrees from fixation) was also reduced for 3 of the 10 subjects. The authors compared batting averages before and after the introduction of the new standards for helmets and concluded that the new standards did not impact on batting average. Therefore, this domain-specific assessment suggests that changes in the visual field do not impact cricket batting. What is not known is whether the players required any compensatory actions to maintain their batting average with the decreased visual field. It should be noted that many other factors can impact batting average. Although the reduced visual field of the new helmet did not appear to affect batting performance, the authors point out that the new standards could affect the performance not just of batters but of any cricketer who wears a helmet (e.g., wicketkeepers). Lastly, the authors referenced studies (described below) that demonstrate that cricket batters make saccades in a downward direction to the location of expected ball bounce and (at least in the case of elite batters) to the predicted location of bat–ball contact. Although still an unresolved matter, if it is assumed that these eye movements are useful in cricket batting and these eye movements are disrupted by the new helmets, then batting may be subtly affected by these helmets. Future studies can be aimed at further understanding both the potential influence of the reduced visual field induced by the new helmet and, more generally, the influence of the visual field on cricket batting and fielding.

#### 3.2.4. Color Vision and the Impact of Ball Color

Another low-level visual attribute that has been examined in cricket players is color vision. These have been domain-general assessments. Because cricket has been played with a red ball against a green background, it could be that deficient color vision may lead to difficulties in playing cricket [34]. There are published studies suggesting that this is indeed the case. For example, it has been shown that the proportion of cricket players with color vision anomalies is less than that of the general population. As mentioned above, Brown and Couper found that only 3% of the cricket players they tested had a color vision deficiency [22]. Further, Harris and Cole evaluated the color vision of 293 cricket players from “seven Melbourne Premier cricket clubs” [35]. Overall, the percentage of players with color vision defects was similar to that of the general (male) population. However, the percentage of players with a severe color vision deficiency was less than that in the population for the highest level (first--grade) players. It might be expected that abnormal color vision would be more detrimental for batters compared to bowlers since batters must “pick up” the ball visually when the bowler releases it. However, color vision deficiencies occurred in similar proportions for the bowlers and for the batsmen. On the other hand, as expected, cricketers with abnormal color vision preferred to field close to the bowler rather than in the outfield. The authors concluded from these correlations that a color vision deficiency is a modest impairment for both cricket batsmen and cricket outfielders.

Scott and colleagues examined slip-catching in five male professional cricketers [36]. Balls were projected from a bowling machine. Red balls and white balls were used under three different levels of illumination. There were no differences in performance associated with ball color or light level. The visibility of pink cricket balls has recently been examined, as this ball is now used for matches that begin during the day and extend into the night. The pink ball is used instead of a white ball, which discolors during use. Adie and Arnold demonstrated that the visibility of the pink ball may be significantly reduced at sunset [37]. In another study, Maguire and colleagues sent a survey to “international and first-class male cricketers” in England and Wales in which questions were asked regarding the visibility of the pink cricket ball [38]. The survey respondents reported that the pink ball was less visible both in fielding and batting compared to the red ball, and that visibility was worst at dark as predicted from the results of Adie and Arnold. The authors concluded that it would be reasonable to take a break at dusk during day/night matches.

### 3.3. Higher-Level Visual Skills

#### 3.3.1. Reaction Time and the Dominant Eye

Reaction time is another skill related to vision that has been examined in cricketers. Reaction time can be considered a higher-level skill, in that it requires the respondent to direct their attention to and respond to a visual stimulus. Early studies suggest that the reaction time of cricketers may be no better than the overall population. The reaction time of Sir Donald Bradman, an international cricketer, was found to be slightly less than that of an “average university student” [39]. Sanderson and Holton found no correlation between the end-of-season batting average of 24 male cricket batsmen in the Liverpool District and both simple and choice reaction time [40]. In a recent paper, Barrett and colleagues examined simple reaction time when a visual stimulus was presented either at the fixated location or 7.5 deg to the left or right of fixation (domain-general assessment) [41]. Simple reaction time is measured with a single stimulus and single response, and was referred to by Barrett et al. as “visually mediated simple reaction time” or VRT. The authors point out that shorter reaction times would likely be of value for athletes. However, they go on to say that the results of those studies addressing a potential relationship between athletic performance and simple reaction time are mixed. Barrett and colleagues suggested that one reason for shorter visual reaction times in athletes could be that they have better gaze stability than nonathletes. Gaze stability in this case refers to the ability to limit blinks and saccadic eye movements around the time that the visual stimulus is presented in the reaction time measurements. Blinks and saccadic eye movements could prolong reaction time because vision is suppressed when they occur. There were five groups of subjects including elite female cricketers from England’s national women’s team, near-elite male cricketers from the “Leeds/Bradford Marylebone Cricket Club University”, elite male rugby players, and a male and female control group. The VRT was significantly shorter for the female cricketers compared to the female controls, and the VRT was also significantly shorter for the male cricketers compared to the male controls. For the rugby players, the VRT was not significantly different between the controls and the players. The authors concluded that gaze stability could not account for the differences in VRT between the cricket players and the controls, and that improving gaze stability through training will probably not to lead to a shorter VRT.

In a different study, Thomas and colleagues measured visual evoked potentials (VEPs) and choice visual reaction time in cricket players and controls [42]. These were again domain-general assessments. Choice reaction time was assessed by asking subjects to strike one of five brass plates, arranged in a pentagon, with a metal rod when these plates were randomly illuminated. They also determined each participant’s dominant eye. The subjects included 25 cricket players (15 batsmen and 10 bowlers) from a cricket training academy in South Africa and 9 “sedentary” male controls. Compared to the controls, the VEP of the players had a shorter N70 latency, suggesting that visual processing was more rapid in the cricket players. However, the choice reaction time was not significantly different between the cricketers and the controls.

Various advantages have been ascribed to the dominant eye compared to the non-dominant eye. One of these advantages is that monocular visual reaction time is shorter when measured with the dominant eye [43,44]. Crossed eye–hand dominance, in which the dominant eye is opposite to the dominant hand, would for many cricket batters, place the dominant eye of a cricket batter closer to the bowler. This could lead to shorter response time and perhaps better batting performance as a result [45]. However, Thomas and colleagues found that crossed dominance was no more common for cricket players than for non-athletes. Similarly, Mann and colleagues conducted a study that demonstrated that male professional cricket batsmen were more likely to use a reversed stance (left-handed stance if right-hand dominant) than inexperienced batsmen [46]. While the results suggested that the reversed stance improves batting performance, the location of the dominant eye relative to the bowler did not affect the chances that a player would be in the professional group rather than the inexperienced group.

#### 3.3.2. Eye Movements—Correlations with Performance

Eye movements can be considered higher-level visual skills. Eye movements have been assessed with both domain-general and domain-specific methods.

In a recent paper by Murray and colleagues, computerized measurements of visual-motor control were made and compared to cricket batting and bowling statistics [47]. Domain-general studies such as this one, in which correlations between ex situ measurements of visual function and on-field performance are made, are common in the sports vision literature [48].

Murray et al. studied 59 Australian male professional cricket players who participated in T20 leagues. T20 is a shorter version of cricket. A total of 30 study participants were bowlers, and 29 were batsmen. The computerized RightEye device (RightEye, Bethesda, MD, USA) was used to assess a number of oculomotor functions including fixations, saccadic eye movements, and pursuit eye movements. Metrics related to the oculomotor variables were calculated by the RightEye device. Oculomotor performance from the RightEye metrics were then compared to batting metrics (runs and strike rate) or bowling metrics (balls, runs, wickets, and ECON (the average number of runs given up for each over bowled)) using multiple regression analyses. Overall, individual RightEye metrics and combinations of these metrics were modestly or highly correlated (R^2^ from stepwise regression analyses ranged from 39.4% to 85%) with batting and bowling performance. The only performance metric that was not related to the oculomotor variables was ECON. Overall, these results are comparable to findings in baseball and other interceptive sports [49,50]. Murray and colleagues concluded that oculomotor variables may impact upon cricket performance, and that training these variables may positively impact performance. Some evidence for these conclusions will be provided in the following sections.

#### 3.3.3. Eye and Head Movements

There have been a number of studies in which the eye and head movements and the gaze (eye + head) of cricket batters have been examined using domain-specific assessments. These investigations have revealed some key differences between expert players and less expert or novice players. There are several reasons why studies of eye and head movements in cricket batters are thought to be important. In general, gaze may be directed toward locations where important information related to performance is gathered. Further, head movements may provide specific advantages in batting as described below. Finally, earlier timing and greater accuracy of predictive saccades that place gaze ahead of the approaching ball may reflect better anticipation of ball trajectory, and these saccades may allow batters to use information after ball bounce to fine-tune interceptive movements.

The first of these eye and head tracking studies was published by Land and McLeod [51]. Vertical eye and head movements were assessed as batsmen hit balls projected from a bowling machine at 90 kph (56 mph). One of the batters was a professional cricket player, while the other two were amateur players. One of these two latter players was described as a “successful amateur”, while the other was described as an “incompetent amateur”. The batsmen tended to make an anticipatory or predictive saccade to a location near where the ball was expected to bounce. Such predictive saccades have been reported in numerous studies in which target interception is required [4,52,53,54,55,56]. The advantages for batting provided by predictive saccades are not clear. Some advantages of predictive saccades that have been suggested include pre-encoding the location of ball bounce and bat–ball contact in eye-centered coordinates, the facilitation of predictions of future pitch trajectories, or deliberately placing the approaching ball in the visual periphery and then using peripheral vision to monitor the trajectory of the ball. This latter idea was recently tested by Vater and Mann, who showed that tracking the ball continuously was adequate to predict when the ball would arrive and that additional peripheral information does not improve these predictions [57].

Following the predictive saccade, Land and McLeod reported that there was a period of relative gaze stability around the time the ball bounced. This was followed after the ball bounce by a period of gaze tracking. The initial predictive saccade occurred earlier for the two better batsmen, and later for the less skilled batsman. In addition, for the “best” batsman, the initial saccade was intermixed with pursuit eye movements.

Land and McLeod did not address the batting performance of their subjects in terms of bat–ball contact, but they did remark that the two better batsmen played “attacking shots” for the short- and full-length deliveries and “defensive” shots for the good-length balls. The other, less skilled batsman played an attacking shot only for the full-length deliveries.

In 2010, Croft and colleagues published a paper on vertical gaze tracking in cricket batting [58]. Gaze tracking data were recorded successfully from nine male cricket players who were attending an “under 19’s squad training camp” in New Zealand. The subjects were all playing in the “senior national leagues for their club sides or for professional teams”. The investigators described the players as sub-elite to elite. A bowling machine was used to project balls at randomized velocities ranging from 61–90 kph (38–56 mph). There was considerable variation in the gaze tracking responses between subjects and importantly, there was no relationship between gaze tracking strategy and ball speed. Four gaze strategies prior to ball bounce were described. One strategy was to pursuit track the ball smoothly while maintaining the image of the ball near the fovea (within 2 deg). A second strategy was to pursuit track the ball initially, then move the gaze below the ball (presumably with a predictive saccade), and finally to return the gaze near the ball prior to the bounce. A third strategy was to place the gaze below the ball (rather than at the ball’s initial release point) and to maintain the gaze at that location until the ball caught up to the eye. Pursuit tracking occurred once the gaze and the ball were co-located. A fourth strategy, termed parafoveal tracking, was to move gaze in the direction of the ball, but to maintain a relatively large (5–10 deg) gaze error.

There was no relationship between the gaze tracking patterns and the percentage of poor bat–ball contacts.

In 2013, Mann and colleagues published a paper on vertical head and eye movements of male cricket batters [59]. Two of the subjects were elite international competitors, and two were high-level club players. A ProBatter machine (ProBatter Sports, Milford, CT, USA) was used to project balls toward the batters. The ProBatter machine includes a screen placed in front of the bowling machine on which a video of a bowler is shown. The video proceeds from the approach of the bowler to the time at which the bowler releases the ball. Thus, the batter is provided with both kinematic and early ball-flight information. The ball is then projected through a hole in the screen. The velocity of the projected balls was 120 kph (75 mph), and the ball lines (directions) and location of ball bounce were varied. The analyses included only trials in which bat–ball contact occurred.

The elite players initially maintained their gaze on the ball or ahead of the ball, while the club players maintained their gaze on the ball or behind the ball. All of the subjects made predictive saccades. The elite batters made earlier (just as Land and McLeod had reported) and larger predictive saccades compared to the club players. The elite batters often produced two saccades (in the good- and short-length trials). One of these was to the predicted bounce point of the ball, and the second was to the predicted location of bat–ball contact. The club batters tended to make just one saccade to the predicted bounce point regardless of ball length. Around the time of the bat–ball contact, the gaze was ahead of the ball for the elite players and behind the ball for the club-level batters.

In terms of head movements, elite batters moved their head with the ball, while the club batters aligned their eyes with the ball. Mann and colleagues suggested that the purpose of rotating the head with the ball was to maintain the ball in a constant egocentric direction relative to the head, thereby reducing the computation of where the ball would arrive at the batter. As mentioned earlier, Regan provided another explanation as to why head tracking might be beneficial in cricket batting (and fielding) [16]. Specifically, estimates of passing distance of an approaching ball were found to be best when the direction of motion in depth was halfway between the eyes.

In 2017, Sarpeshkar and colleagues published a paper in which they examined head and eye movements of 43 male cricket batters in significant depth [54]. A ProBatter ball projection machine was used to project balls toward cricket batters at a velocity of about 119 kph (74 mph). The projected balls varied in length to ball bounce, line or direction, and trajectory (straight or curved). Subjects included adult elite players who competed for “their state or country at a senior level”, a youth elite group who competed for “their state or country at an under 19 or under 17 level”, an adult club group, and a youth club group. The following is a summary of some of the significant results of this study. It should be noted that interactions between the variables of interest were common in these analyses.

In examining the results from the straight trajectory trials (analysis 1), for elite batters, gaze was further ahead of the ball compared to the club batters, as Mann et al. had reported previously [59]. This suggests that elite batters better predict the trajectory of the ball. In terms of head movements, the adult elite batters maintained the same gaze-head angle relative to the ball, while the rest of the batters moved their gaze further ahead of the head when the balls bounced further from the batters. While Mann and colleagues had previously reported that head tracking was more common in elite batters, there was no difference in the head–ball angle for the elite and club players.

Different from the results of Land and McLeod, in which predictive saccades occurred earlier for the more experienced players [51], Sarpeshkar and colleagues reported that the time at which saccades to the predicted location of ball bounce occurred did not vary between their subject groups for the straight trajectories. Sarpeshkar et al. attributed differences between the results from Land and McLeod and the results from their study to the fact that Land and McLeod used a slower ball velocity and more predictable trajectories. On the other hand, just as Mann and colleagues reported [59], elite players more commonly made a second saccade to the point of bat–ball contact compared to the club players. Finally, batting performance (assessed as the “quality of bat–ball contact”) was better for the elite batters compared to the club batters.

In a second analysis, head and eye movements and gaze tracking behaviors were compared under conditions where batters knew that the ball would follow a straight trajectory and under conditions when the ball might not follow a straight trajectory (random trials). In the random trials, changes in gaze behavior occurred that the authors described as “novice-like”. Specifically, the gaze was further behind the ball, there were fewer predictive saccades, and those saccades that were predictive occurred later, and the gaze at bat–ball contact was less commonly in the direction of the ball when the bat contacted the ball. These findings are interesting and similar to those from Sarpeshkar and colleagues [15] and those cited earlier on batter kinematics [14], because changes in behavior were not necessarily driven by the (swinging) trajectories but rather by the possibility that the trajectory may not be straight. Interestingly, the quality of bat–ball contact was negatively impacted to a greater extent for the elite batters compared to the club batters when the possibility existed that the ball may follow a swinging trajectory. This decrease in performance for the elite batters resulted in equivalent quality of bat–ball contact between the elite and club batters.

A third analysis compared gaze tracking when the batters attempted to hit balls that swung versus balls that did not swing. Differences in the behavior of elite and novice batters were exaggerated in the swinging trials compared to the straight trials in that elite batters were better able to maintain their gaze in the direction of the ball in the swinging trials. The frequency of predictive saccades to the location of ball bounce was similar for the swinging and straight trials. However, only the elite players were more likely to make oblique saccades in the swinging trials. The quality of bat–ball contact was reduced when batters attempted to hit swinging trajectories.

Finally, in a fourth analysis, Sarpeshkar and colleagues compared visual-motor behavior when projected balls swung toward the batter (in-swing) and when these balls swung away from the batter (out-swing). The batters’ gaze was less likely to be ahead of the ball when the ball swung out. While the frequency of predictive saccades did not depend on the direction of ball swing, batters made earlier saccades in the out-swinging trials that the authors suggested were the result of the batters mistaking the out-swinging trajectories for straight trajectories. The quality of bat–ball contact was reduced for the out-swinging trajectories compared to the in-swinging trajectories.

In summary, there is variability in the eye and head movement and gaze tracking findings in these studies [51,54,58,59]. It seems that expertise influences the results, as does the predictability and perhaps the velocity of the approaching balls. Specifically, experts tend to maintain their gaze further ahead of the ball, to make more and earlier predictive saccades to the locations of ball bounce and bat–ball contact, and to more commonly place their gaze at the location of bat–ball contact. Predictable ball trajectories and slower ball velocities lead to earlier predictive saccades. These results may suggest that training for cricket batters could be directed at teaching non-elite players to emulate the gaze tracking strategies of more advanced players. However, because the influence of gaze tracking and eye and head coordination on batting performance is not entirely clear, future studies are necessary to determine whether gaze tracking strategies result in better batting. Alternatively or in addition to eye movement training, because predictive saccades are thought to reflect anticipation, it may be that training anticipation could improve performance in cricket [60]. However, none of these eye and head tracking studies included assessments of the relationship between eye and head movements, lower body movements, and early portions of the bat swing. According to the two-stage model of interception proposed by Morris-Binelli and Müller, lower body and early bat swing movements are directly related to anticipation [7]. Future eye tracking studies should include assessments of lower body and early bat movements to determine whether eye movements early in the ball’s flight and the timing and accuracy of predictive saccades reflect anticipation in the same way as lower body movements.

## 4. Vision Training and Cricket Performance

The impact of training upon cricket has been explored in a number of studies. These studies, summarized in Table 1, vary substantially both in the training methods used and the playing level of the participants.

In 1996, West and Bressan conducted a study and assessed the impact of two types of visual training on the ability of cricket batsmen to judge the length-of-ball [61]. Selected visual skills were assessed and trained in a domain-general manner. Some of the training was directed at eye movements, which are thought to be related to performance in cricket matches [24,47]. In addition, some of this training could improve stereoacuity [62], which Barrett and colleagues found to be better in elite cricket players compared to the general population [9]. Local male cricket players aged 19–24 were divided into three groups, with 12 players receiving general visual skills training (accommodative flexibility, convergence in 9 directions of gaze, convergence and divergence, horizontal saccades, vertical saccades, rotations whereby participants tapped on letters placed on a rotating disc, and ocular-motor programming), 12 players receiving visual training on skills thought to be specific to batting (2 convergence/divergence exercises and 1 accommodative flexibility exercise), and 12 players acting as a control. All 36 players completed pre- and post-training assessments including the following: the time required to find letters on a rotating disc (rotations), the efficiency of horizontal and vertical saccades as participants read horizontally displaced or vertically displaced pairs of letters, the accommodative facility using plus–minus diopter flippers, and the ability of each player to judge the length-of-ball of balls projected toward them using a bowling machine and using a screen that blocked the players’ view of the final 4 yards of the ball’s approach. After three weeks of training, the length-of-ball judgments were statistically improved in both groups that completed the visual training. The group who underwent general visual training showed statistically improved scores for all of the visual tests performed before and after training, which should not be surprising, since the tests measured the same skills used in the training. The only significant improvement noted for the batting-specific visual training group was found for accommodative flexibility, which was one of the training skills used over the previous 3 weeks. The control group showed no improvements in the visual skills or the judgments of ball-length, which likely shows that visual skills improved if the participants completed training specifically for that skill. The most important outcome of this paper that supports visual training for cricketers is the improvement in judging the length-of-ball with visual training. The results suggest that domain-general training can positively impact domain-specific assessments. While (uncoupled) judgments of ball-length were examined in this study, further research should include pre-training and post-training assessments of (coupled) batting performance, as uncoupled and coupled responses are known to vary [30,63].

A study by Stretch utilized 16 cricketers at a cricket academy (referred to as batsmen and bowlers) aged 17–19 years, who were divided into an experimental and control group [64]. The players utilized a bat with electrodes covering the surface of the bat to detect the location of contact between the ball and the bat, and a bowling machine set to 100 kph (62 mph) was used, so that all strokes could be played off the front foot. Batting performance was assessed before and after three weeks of vision training was completed four times per week for the experimental group. Then, the experimental and control groups were switched, apparently such that the control received vision training. The vision training consisted of a rotator pegboard, the wall-mounted AcuVision 1000 eye–hand coordination device, and various hitting skills (up and down on a bat, against a wall with a bat, hitting a suspended swinging ball), and tossing and catching (juggling, underarm beanbag toss into a box, target throwing). While these training exercises were largely directed at eye and hand coordination and these skills have been found to correlate, for example, with batting metrics in baseball [65], the authors concluded that vision training did not result in larger improvements in hitting performance compared to conventional hitting practice. In this case, a combination of domain-general and domain-specific training did not yield improvements in a (coupled) domain-specific assessment.

A study by Balasaheb and colleagues examined 30 competitive, club-level male cricket batsmen aged 16 to 25 [66]. Participants were divided into 3 groups of 10, with an experimental group completing visual training exercises for 30 min a day, three days per week, for six weeks. The exercises included swinging target exercises (Marsden ball and swinging ball with pointed finger, depth perception training, drills of reaction time, Hart chart therapy, alternating fixation on distance and near charts, juggle stick exercises, and vision ring exercises). A placebo group of 10 participants were given reading material and watched cricket matches for 6 weeks, and the control group did not have any assignments beyond the daily practice sessions that all 3 groups attended. Testing of reaction and movement time, depth perception, saccadic movements, accommodation, and batting performance (batting average) were assessed before and after the treatment period and were compared for each group. In the experimental group, all tests revealed statistically significant improvements. The placebo group analysis revealed statistically significant improvement in movement times, ocular motility, and depth perception tests; and the control group analysis found statistically significant improvements in movement time to the right, ocular motility tests and depth perception. Many of the tests improved in all groups. Batting averages from five games before training and five games after training also showed significant improvements for all three groups, also with a higher significance level for the experimental group found with ANOVA and post hoc analysis. One positive attribute of this study is that it evaluates playing metrics (batting) before and after the training, rather than only evaluating vision exercises. Once again, the results suggest that domain-general training methods can positively impact on (coupled) domain-specific assessments. The small group sizes used in this study were a good first step in collecting information to use for sample size calculations in future, larger studies. The improvements in the placebo group and the control group emphasize the need for inclusion of such groups in clinical trials in sports vision to properly assess the impact of vision training methods on performance [48].

**Table 1:** Summary of vision training studies in cricket.

Study	Number of Participants	Player Status	Player Ages	Type of Training	Type of Testing	Results
West and Bressan [61]	36 (3 equal groups)	Local players—batsmen	19–24	Group 1: General visual skills training Group 2: Visual training specific to batting Group 3: No training	Ability to judge length-of-ball (occlusion/anticipation testing)	Both groups that completed training had improved judgement of length-of-ball after training.
Stretch et al. [64]	16 (2 groups)	Attendees of a cricket academy	17–19	Part 1: Group 1: Vision training Group 2: No vision training Part 2: Groups alternated training	Batting as assessed using a bat with electrodes to detect impact location and a bowling machine	There was no difference in batting accuracy or consistency when comparing the groups that completed vision training to the group that did not complete vision training at post-test 1, or after both groups had training and were rested at post-test 2.
Balasaheb et al. [66]	30 (10 per group)	Competitive club level	16–25	Group 1: Visual training skills Group 2: Placebo video viewing and reading Group 3: No added training	Visual skills and batting average	All groups had improved batting.
Calder and Kluka [67]	30 (2 equal groups)	High school with at least 3 years experience	13–19	Group 1: Computer-based vision training Group 2: Placebo training	13 visual skills and 6 sport-specific skills	Throwing accuracy and directionality were improved from pretesting; throwing distance was improved and significantly better than the control group.
Kruger et al. [68]	13	Under 19	Under 19	Visual skills tests/training with running between skills	Implied to be the same tests as used in the training	Many visual skills that were trained improved over 8 weeks. No cricket skills were assessed.
Hopwood et al. [69]	12 (2 groups)	Senior level	18–26	Group 1: On-field training (5) Group 2: On-field and perceptual training (7)	Occlusion and anticipation to assess fielding	No significant improvement in time to initiate movement in both groups. Improvement in fielding success in those who completed perceptual training.
Edgar et al. [27]	36	18 cricket players and 18 non-cricket players	17 or older	Group 1: 2 additional dynamic visual acuity assessments served as training Group 2: No training	Dynamic visual acuity assessments	Dynamic acuity improved in the people who had additional training. No cricket skills were assessed.
Shunmuganathan [70]	36 (3 equal groups)	Under 19	Under 19	Group 1: Cricket skills and visual training Group 2: Cricket skills training only Group 3: No additional training	Batting performance (not defined)	Players in the cricket skills training group and cricket skills with visual skills training both showed improvements in batting performance (batting metrics were not described).
Wimshurst et al. [71]	24 (4 equal groups)	County level	Mean 24.38 ± 3.29	Group 1: Practical visual and visual coordination drills Group 2: Online visual training Group 3: Nintendo Wii games Group 4: No vision training	14 visual skills and 7 cricket skills (including batting, catching and throwing)	All groups improved in skills. ANOVA found a significance when comparing the pre-training test to the post-training test for all data, with no significant differences in the groups themselves.
Brenton et al. [72]	12 (2 groups)	State cricket level	Mean age 23.5 ± 2.75 and 22.2 ± 3.01	Group 1: Visual-perceptual training (point-light display and temporal occlusion) Group 2: Control	Temporal occlusion with batting stroke to anticipate the oncoming ball	Anticipation improved in the training group but not in the control group. Batting average was higher during the experimental season in the training group compared to controls.
Brenton et al. [73]	39 (3 groups)	District club level	18–36	Group 1: Visual-perceptual training (included use of temporal occlusion) Group 2: Visuomotor training (included use of temporal occlusion) Group 3: Control	Temporal occlusion with stance action to anticipate the oncoming ball	The groups with training performed better on the post-test than the pre-test. The visuomotor group tested better than guessing for all scenarios, and the visual training group performed better than guessing for the ball release scenario. No on-field metrics were analyzed.
Kumar and Kadhiravan [74]	30	Club level	16–24	Unknown visual skills and batting assessments	Visual skills and batting performance (not defined)	Statements of improved visual skills and “batting performance” are made but not explained.

A study by Calder and Kluka evaluated 30 male high school cricket players with at least three years of experience, dividing the participants into two groups of players with matching numbers of participants who played each position [67]. One group served as a control and completed placebo “training,” while the other group used computer-based vision training software (EYETHINKSPORT) which appears to have involved both domain-general and domain-specific training methods (http://www.sportsci.org/news/news9705/hockeyvision.html, accessed on 9 August 2023). The training was completed for three sessions per week for four weeks. Assessments of domain-general visual skills tests (including accommodative flexibility, horizontal and vertical saccades, depth perception, rotational skills, and eye–hand coordination) and domain-specific cricket skills tests (reaction time in catching, speed and accuracy of catching, peripheral awareness and catching, directional throwing accuracy, distance throwing accuracy) were completed before and after the software was used. Those using the computer training showed significant improvements between the pre- and post-assessments for 13 visual skills and six sport-specific skills. Speed and accuracy of catching was statistically different when comparing the improvement from pre- to post-testing and the experimental group compared to the control group. The experimental group had a statistically higher number of successful catches when comparing pre- to post-training, although the improvement was not statistically better than in the control group. Throwing accuracy, directionality, and distance were improved statistically when comparing pre-testing to post-testing in the experimental group. Comparison of throwing distance between the experimental and control groups was also significantly different. Batting was not assessed in this study. The use of cricket (domain-specific) skills in the assessments before and after vision training were useful. Further testing with a larger study population would add more validity to the findings in these small groups.

A study by Kruger and colleagues recruited 13 under-19 cricket players to complete an 8-week visual skills training program that included two training sessions each week [68]. Whether the participants were male or female was not made clear. The 60 min training sessions included 15 to 20 min of running, and visual testing was interspersed in the session. The explanation for this protocol was that increasing the heart rate would increase fatigue and facilitate visual testing under stress. It was believed that this strategy would improve visual concentration, reaction time, speed “of the mark”, ability to track a moving ball, and “accuracy and reflexes”. Visual skills tests included the following: accommodative flexibility (rock), Randot stereo acuity, eye tracking pursuits using a rotator pegboard (time required to put pegs into holes adjacent to specific letters on a rotating pegboard wheel with letters alphabetically arranged circumferentially), X-chart saccadic testing (time to complete reading letters horizontally separated into two columns from top of chart to bottom), peripheral awareness and responses (Wayne Membrane Saccadic Fixator), a hand/eye coordination task that involved throwing a ball with one hand toward a wall and catching it with the other hand with the number of successful catches assessed, a ball catching skill test (modified Crucifix ball drop), a visual recognition computer test in which a sequence of four colors was recalled, visual anticipation computer game in which the user keeps a “ball” on the screen by manipulating a paddle to prevent it from hitting the side, accuracy testing using a computer game that involves quickly clicking on red balls as they appear on a screen while using a mouse, and a color vision assessment in which a participant must draw what is seen on 9 cards designed to find red-green color deficiencies. While not explicitly stated, it appears that the pre-training and post-training domain-general assessments were the same tests that were used in the training. Accommodative flexibility, hand/eye coordination, peripheral awareness, ball drop skills, eye tracking, visual anticipation, and accuracy skills all showed significant increases in scores. The results of this small study reveal that taking the tests frequently improved the scores. While it is known that visual skills such as eye movements and visual anticipation are likely related to improved performance in cricket [20,24,54,59], no conclusions were made in this study regarding whether these improved skills translate to better cricket playing skills.

The occlusion technique was used in a training study by Hopwood and colleagues. Twelve senior level male cricket players completed in situ and perceptual testing of fielding before and after six weeks of training [69]. Training sessions included on-field training by the players’ coaching staff for five participants, and on-field and perceptual training three times each week for the other seven participants. The perceptual testing and training utilized life-sized videos of batters filmed from the perspective of three fielding positions, with black video frames blocking the moment of ball and bat contact and participants predicting the direction the ball would have taken when hit. Those participants who only completed on-field training had a decrease in accuracy in anticipating the direction of the ball, while those with perceptual training improved significantly in anticipatory accuracy from the pre-test score. The perceptual training group had a greater improvement in fielding success after the training, but neither group had a significant change in the meantime to initiate movement when comparing pre- and post-testing. The authors concluded that (domain-specific) perceptual training is beneficial for fielding with this very small sample size when participant responses were both uncoupled and coupled. Larger studies would provide more compelling evidence for this conclusion.

Edgar and colleagues assessed dynamic visual acuity in 18 male cricket players under the age of 19 and 18 male non-cricket players [27]. While this study was mentioned previously in this manuscript, the training aspect of the study is discussed here. A Landolt C (sized 6/15 m or 20/50 feet) with crowding bars was presented, moving on a screen at various speeds for 600 msec at an initial visit and at a final visit 7 weeks later. Some participants completed domain-general training by repeating the tests two additional times between the initial and final tests. There was no significant differences between the cricket players and non-cricket players, or between the positions of the cricket specialties (bowlers, batters, and all-rounders). Of those participants who attended the two additional training visits, dynamic acuity was significantly improved compared to those without additional training visits. Interestingly, the mean dynamic acuity improved with each additional test/training visit, with significant differences between the first visit and third visit and first visit and final visit. This improvement was also seen in the group that did not complete additional training visits. The results show that dynamic acuity can be improved by training, which might be expected to improve cricket play, but no conclusions regarding cricket playing performance could be drawn.

A study by Shunmuganathan evaluated batting performance in 36 male cricket players, dividing them into three groups: one group that completed skills training and visual training, one group that completed only skills training, and one group that served as a control with no additional training [70]. The two experimental groups both had statistically significant improvements in batting before and after 12 weeks of training 3 days per week, while the control group did not have statistical improvement. Further analysis of the post-training scores found a statistical difference between the group that had skills training with visual training compared to the group that only had skills training. There is no description of how the author defined visual training nor are there any examples of the types of training used in the study. While the outcome appears positive, it is difficult to draw any conclusions based on the limited information given in this manuscript.

Wimshurst and colleagues conducted a study of 24 male county cricket players divided into four “training” groups who participated in six weeks of training [71]. One group completed practical drills (reaction ball, juggling, pencil push-ups, pursuits, juggling and kick a football, number/letter trace, Brock string, carton catch, peripheral catch, punching O’s, balancing catch, double Brock string), one group completed online training (eye movement speed, peripheral awareness, flexibility of focus, eye tracking, eye jumps, 3D viewing), one group played Nintendo Wii games (shooting range, Find Mii, table tennis, Pose Mii, various sub-games in the Mario and Sonic at the Olympic Games game, and various sub-games in the Wii Fit game), and a final group served as controls and participated in activities including rebound slip catch, rebound net, intercept and throw, and throw to target. Domain-general visual tasks (Howard Dolman, rotator board, horizontal saccades, focus flexibility, crazy catch, crucifix ball drop, visual memory, Wayne saccadic fixator (9.1, 9.11, 9.21, 9.62), remote control car test, Bassin Anticipation Timer, flippers) and domain-specific cricket related tasks (bat to over, bat to mid, bat pull, bowl Yorker, diving catch, high catch, throw to stumps) were used to assess skills before and after the training. Overall, all of the training groups improved in the vision and cricket related tasks, while the control group showed no significant improvement. However, this improvement did not occur in all of the tests. The analysis did not find a statistically significant difference between the training groups, although the authors indicate that the improvement in visual and cricket skills was larger for the practical group compared to the computer-based training groups.

Brenton and colleagues performed a study in which twelve “emerging” cricket batsmen from an Australian state cricket squad were included. Six of the subjects received visual-perceptual training, and six subjects served as controls [72]. The training consisted of temporal occlusion of a point light display of a bowler in which only advance information was presented, and participants were required to play an appropriate batting stroke. The training group also practiced mimicking the bowler’s motion. Anticipation was improved in the training group but not in the control group. Interestingly, during the season in which the training occurred, the batting average of the training group was moderately improved compared to the season before the training. The batting average of the control group during the season in which the experiment was performed decreased slightly from the season prior to the experiment. In addition, the batting average of the training group was higher than that of the control group during the time in which the study was performed.

Brenton and colleagues also completed a study of 39 district club level specialist male batters aged 18–36 [73]. Participants were divided into three groups, with the first completing visual-perceptual training, the second group completing visuomotor pattern training, and the third group serving as a control. In all conditions, participants were required to play a batting stroke appropriate for the predicted ball type. After an initial pre-test in which participants viewed videos of actual bowlers, both of the training groups received domain-specific temporal occlusion training in which the participants viewed a point-light display (lights representing the motion of a fast bowler) of three ball types: a full-length outswinger, full-length inswinger, or short ball. The occlusion of the point-light display occurred at key moments (prebounce occlusion, ball release occlusion, or no occlusion) during the presentation. The visual-perceptual and visuomotor groups completed the occlusion training, but in addition, the visuomotor group was also given a cricket ball after each presentation to bowl using the same motor pattern as that used in the video that they had just viewed. All groups completed testing before and after the four-week training period, which included two training sessions per week. Overall, all three groups performed similarly on the pre-tests, with none of the groups identifying the ball type better than guessing. The two groups that completed training showed significantly better performance than guessing in the post-testing, suggesting that domain-specific training positively impacted on the (coupled) domain-specific performance assessment.

Kumar and Kadhiravan completed a study that assessed changes in visual skills and batting performance of male cricket players who underwent sports vision training for 12 weeks [74]. Thirty club level cricketers completed the sports vision training for 30 min, three times each week. The premise of that study looks promising, in that game skills are assessed; however, the methodology was not explained, and the testing and training assessments and batting performance scale are not presented. The authors claim that *t*-tests were performed, but the results of statistical tests are not given to support their statements that eye/hand coordination, eye/foot coordination, visual reaction time, depth perception, dynamic acuity, or batting performance are “significantly” improved.

### Summary of Cricket Training Studies

The criteria for evaluating the quality of clinical trials in sports vision were described recently and can be applied in assessing the studies in this manuscript [48]. These criteria include appropriate inclusion of study controls and inclusion of adequate sample sizes to provide adequate study power. While many of the studies described in the current paper do include a control or placebo group, the studies generally involve very small sample sizes. Sports vision studies often involve small samples, and this is at least partially attributable to the difficulty in recruiting and scheduling athletes to participate in these studies. These issues are particularly problematic when recruiting elite athletes as study participants. On the other hand, Müller and colleagues have pointed out that as the level of expertise increases, this necessarily reduces the potential sample size of participants [75]. Thus, a small sample size in a study involving athletes at the highest level is representative of that population, and increasing the sample size in a study by including athletes at many skill levels may not reflect the behavior of the most expert athletes. In another paper, Müller and colleagues used chronometric movement analyses to measure the behavior of eight cricket batsmen from an Australian “state high performance squad” [76]. While some of the batsmen varied in the biomechanics of the swing, the frequency of bat–ball contacts was similar between the batsmen. This suggests that the examination of individual responses rather than aggregate responses could be important in sports vision studies. Lastly, it has been suggested recently that linear mixed-effects modeling can be applied in analyzing the results of studies with small sample sizes [77]. The training studies described in the current paper also vary substantially in the training methods used. In 11 of the 12 studies, there was improvement in visual skills, judgments related to cricket, batting or catching performance, or in more than one of these measures. However, a domain-specific post-training assessment was not always included, and this is critical in evaluating the efficacy of the training method. Overall, these results are promising, but controlled studies with larger populations of athletes including both males and females are needed.

## 5. Conclusions and Future Directions

Visual skills that have been discussed in this paper can be divided into high-level and low-level skills based on the classification scheme provided by Hodges and colleagues [3]. Some, but not all of the fundamental and lower-level visual skills are similar between cricket players and the general population and between cricket players at different levels. Specifically, visual acuity [9], ocular alignment as assessed with the heterophoria [22], and possibly the visual field [33] do not appear to be better or, in the case of the visual field, do not appear to influence performance in cricket players. The lack of impact of visual acuity (i.e., blur) and the visual field on cricket performance has been demonstrated with domain-specific assessments. On the other hand, stereopsis may be associated with cricket performance, but this conclusion is based on a domain-general assessment. Disrupted binocular function has been found to negatively influence baseball batting [78], but a domain-specific assessment of the impact of stereopsis on cricket batting has not yet been performed. Finally, color vision has been assessed in a domain-general manner, and color vision deficits appear to have a moderately negative impact on cricket performance [35]. Domain-specific assessments of the impact of color vision on cricket performance are warranted.

In terms of higher-level visual skills, there is evidence from one study that simple reaction time may be shorter in cricket players [41], but in another study, the choice reaction time did not vary between cricket players and non-players [42]. Both of these latter studies used domain-general assessments. On the other hand, there is (domain-specific and domain-general) evidence that conjugate eye movements vary between elite and sub-elite cricket batters [24,54,59], although one study that employed domain-general subjective measurements of conjugate eye movements and measures of vergence eye movements (near point of convergence) concluded that there was no difference in the percentage of cricket players with abnormalities in these eye movements compared to the general population and no difference when players at different grades were compared [22]. Future studies can be directed at understanding whether training sub-elite batters to emulate the eye and head movement and gaze tracking behaviors of elite batters can improve on-field performance, or whether methods aimed at improving anticipation can improve both eye movements and cricket performance. Finally, domain-specific temporal occlusion studies consistently suggest that cricket players have more rapid and more efficient attentional and anticipatory processes.

A question of interest is whether the just-described findings on the significance of various visual skills can inform the vision training methods employed in cricket. In a recent paper on sports vision training, Poltavski and colleagues [79] reviewed papers in which sports-related visual skills were divided into hardware and software skills. As detailed by Poltavski et al., hardware skills include static and dynamic visual acuity, depth perception, accommodation, fusion (convergence), color vision, and contrast sensitivity [80]. The software system includes eye–hand coordination, eye-body coordination, visual adjustability, visual concentration, central-peripheral awareness, visual reaction time, and visualization [79,81]. Any advantages of hardware skills in cricket have all been demonstrated using domain-general assessments, and for many of these skills, no advantages were demonstrated. On the other hand, advantages for the software skills examined so far have mostly been demonstrated using domain-specific assessments, and in all cases, these software skills were found to positively impact on cricket performance. The one exception is reaction time, which has been assessed in a domain-general manner and for which the results are mixed in terms of whether reaction time is better in cricket players compared to non-players. This is not to suggest that training of hardware skills should not be a part of the training regimen. In fact, there are two studies described in this paper in which domain-general training regimens focused on hardware skills resulted in better performance in a domain-specific assessment [61,66]. The point is that (domain-specific) training of software skills is generally successful (see Stretch et al. [64] for an exception) and should be included in the training regimen for cricket players.

An interesting point to consider going forward is that while studies on the advantages of hardware skills in cricket are equivocal thus far, in many of the training studies, the vision training includes or is focused on hardware skills such as accommodation and vergence eye movements. If training these visual skills improves performance in cricket, then it may be that the training results in supra-normal lower-level visual skills, or that the training results in improvements in related skills such as attention in addition to changes in the visual skills that are trained and tested.

In spite of the diverse levels of the cricket players in the studies discussed here, the conclusions were often similar. However, it is possible that the visual skills or visual behaviors of the most elite cricket players vary from those of even near-elite players [51,59]. Similarly, there are very few studies cited in this manuscript that include female participants. Lastly, while those visual training studies that have been performed thus far have in many cases yielded positive results, future clinical trials should incorporate larger and more diverse groups of participants and the outcome measures should involve performance-based hitting and fielding (domain-specific) outcome measures and assessments of individual differences in response to different combinations of training methods.

## Data Availability

No new data were created in preparing this manuscript.
